# Optical Light Sources and Wavelengths within the Visible and Near-Infrared Range Using Photoacoustic Effects for Biomedical Applications

**DOI:** 10.3390/bios12121154

**Published:** 2022-12-10

**Authors:** Unsang Jung, Jaemyung Ryu, Hojong Choi

**Affiliations:** 1 Production Technology Research Center, Kumoh National Institute of Technology, 61 Daehak-ro, Gumi 39177, Gyeongsangbuk-do, Republic of Korea; 2 Department of Optical Engineering, Kumoh National Institute of Technology, 350-27 Gumi-daero, Gumi 39253, Gyeongsangbuk-do, Republic of Korea; 3 Department of Electronic Engineering, Gachon University, Seongnam-daero, Sujeong-gu, Seongnam 13420, Gyeonggi-do, Republic of Korea

**Keywords:** photoacoustic effect, optical source, wavelength, photoacoustic material

## Abstract

The photoacoustic (PA) effect occurs when sound waves are generated by light according to the thermodynamic and optical properties of the materials; they are absorption spectroscopic techniques that can be applied to characterize materials that absorb pulse or continuous wave (CW)-modulated electromagnetic radiation. In addition, the wavelengths and properties of the incident light significantly impact the signal-to-ratio and contrast with photoacoustic signals. In this paper, we reviewed how absorption spectroscopic research results have been used in applying actual photoacoustic effects, focusing on light sources of each wavelength. In addition, the characteristics and compositions of the light sources used for the applications were investigated and organized based on the absorption spectrum of the target materials. Therefore, we expect that this study will help researchers (who desire to study photoacoustic effects) to more efficiently approach the appropriate conditions or environments for selecting the target materials and light sources.

## 1. Introduction

Photoacoustic effects are generated by acoustic waves resulting from the interaction between light and target materials, which depend on the thermodynamic and optical properties of the materials and the properties of the incident light [1]. Therefore, photoacoustic technology involves measuring the magnitudes of ultrasonic (acoustic) signal outputs according to the light energy absorbed by the target molecules.

The first photoacoustic technology was voice transmission, developed by Bell in 1880 [1]. In modern times, with the development of laser technology, pulsed lasers with high peak energy (concerning power) are generally used under a 100 ns pulse width and some studies have been conducted on photoacoustic technology through modulation driving in the continuous method [2,3]. In addition, a light source capable of obtaining high energy with a lower output is also under research to increase the contrast and reduce harm to sensitive objects that may be damaged by the exposed energy, such as living tissues [2,3].

The commercially available and widely applied 532 or 1065 nm nanosecond lasers and optical parametric amplifiers (OPAs) or dye lasers that can selectively usable specific wavelengths are widely used as light sources [2,3]. Low-cost light sources and selected single wavelength/energy light sources are also under development for commercialization. Recently, there has been a growing trend to use low-cost laser light sources owing to their economic advantages. Thus, many attempts have been made to develop photoacoustic technology using a modulation mode based on laser diode (LD) or light-emitting diode (LED) light sources [4,5]. However, laser light sources can still produce wavelengths of various spectra compared with low-cost LD or LED light sources [4,5]. In addition, laser light sources with relatively high energies are still efficient in obtaining depth or signal contrast, especially for discovering new and unknown applications.

There are two types of photoacoustic irradiation methods: pulsed and continuous (CW). One of the most important parameters in the pulsed photoacoustic type is the resonance between the photoacoustic frequency of the target material and the pulse duration of the incident light [6,7]. The CW photoacoustic type is related to the energy absorption rate according to the wavelengths of the molecule concerning the light energy incident on the targets [6]. If we consider other influencing aspects, the differences between the ultrasonic signals produced by the object can help obtain contrast signals or images based on the optical spectra of different molecules generated according to the incident light energy and wavelength absorption, pulsed, CW irradiation type, and pulse duration [8]. The optical energy absorption rate is different for each material and wavelength [6]. As the methods of expressing these absorption properties, there are absorption rates, absorption coefficients, molar extinction coefficients, and absorption spectrums [6]. Examples of these spectroscopic research results include studies on the optical properties of water [9], rat blood samples with induced hyperglycemia [10], and studies on the optical properties of human skin [11], which are important variables in research on photoacoustic technology.

The photoacoustic spectroscopy technology using white light sources or broadband light sources is excellent for studying the absorption spectrum of specific materials, analyzing the specificity of the materials, and selecting a range of target wavelengths [6]. However, compared to the cases when using a single wavelength or selective wavelength combination for practical applications, the performance when using the specific contrast of the target material may be reduced because the optical outputs are generally weak or it is difficult to sufficiently narrow the bandwidth [8]. In addition, the light energy absorption spectrums for the application of photoacoustic effects are important in the selection of wavelengths for the target materials because the wavelengths are determined according to the absorption spectrum peak, and the required light sources are selected to obtain the suitable sensitivity to detect the target materials [8]. For example, a certain wavelength is designed in terms of photoacoustic imaging by the absorption peaks or ratio for specific molecules, certain absorption peaks, or ratios for certain molecules [6]. Conversely, light sources with a selected wavelength can also be used as contrast media by finding the target materials that can be detected from the light source in possession.

To generate the ultrasonic signal outputs from photoacoustic systems, sufficient light energy needs to be irradiated to generate a broadband photoacoustic wave of tens or hundreds of MHz [12,13]. In the case of photoacoustic microscopy (PAM), the detection sensitivity of photoacoustic signals is affected by the incident laser light fluence, imaging depth, optical wavelength, absorption cross-section of the target, and detection efficiency of the ultrasonic transducer [8]. The incident intensity of the light source is typically lower than the saturation intensity; therefore, the detection sensitivity is proportional to the laser light fluence [8]. However, the laser fluence decreases dramatically as the imaging depth of the target increases [14]. If the light sources are easily considered, except for conditions such as converter detection efficiency or cross-sectional area, a CW or pulsed laser with high fluence and the wavelengths of light sources having sufficient absorption spectrum in the target materials need to produce the appropriate outputs [8]. To obtain the appropriate signals, we need detailed information on the incidence of sufficient light energy, the selective wavelengths that produce ultrasonic signal outputs from the target molecules, and the characteristics of the incident light energy (pulsed, CW, and duration of incident light). In 2018, Huabei Jiang described the experimental method for optical energy and photoacoustic technology at wavelengths for the acquisition of photoacoustic signals [15]. This paper describes the applications of photoacoustic effects according to the light sources, wavelengths, specifications, and methods.

In terms of hardware, in the application of photoacoustic technology in the industry, faster signal acquisition is required owing to changes or movements, as in the case of a moving target or living tissue, not a fixed target. Thus, research on improving imaging speed is being actively conducted. In photoacoustic technology, the factors affecting imaging speed include the laser pulse retention rate, scan speed, and computing power, and the most important factor among these is the high laser repetition rate [16]. However, because the acoustic signals generated by photoacoustic effects are slow in signal processing with a propagation speed of 1500 m/s, it is necessary to optimize the appropriate laser, scan speed, and computing power according to the purpose of use rather than unconditionally improving the speed.

The fields of use of the photoacoustic effects at each wavelength are briefly mentioned here. The light sources of the photoacoustic systems used for eye imaging are mainly at 532 and 1064 nm, whereas two different wavelengths, 570 and 578 nm, are selectively used for oxy-hemoglobin and deoxy-hemoglobin of the blood [17]. In blood or blood vessels, there are many applications near 400 nm (microscopy type) and near the 500 nm range, which are visible light spectrum ranges that indicate the maximum absorption of hemoglobin [18]. For near-infrared (NIR) ranges, the optical absorption coefficient is lower than that of visible light, but scattering and absorption are lower than that of visible light; therefore, penetration depth can be added [16]. In addition, there is a relatively high output in the NIR range, and these wavelengths are widely applied for tissue imaging of not only blood vessels but also deep areas [4]. For breast cancer, the tumor is identified with multiple wavelengths, using a 1064 nm wavelength for the deep area and a wavelength of approximately 757 nm to increase the specificity of the tumor [19,20]. In addition, the optical absorption of a particular substance is identified and applied as a contrast agent or treatment for the diagnosis, treatment, or determination of the presence or absence of substances or tumors based on the photoacoustic signals of the substance [16]. 

In this paper, we analyzed published photoacoustic-related papers obtained in the Google Scholar and PubMed search engines, focusing on optical sources, such as commercially available lasers that selectively use wavelengths and self-developed light sources. In addition, the characteristics and performance of the light source essential for photoacoustic technology and the reasons for determining the wavelength of the light source in the application are described.

## 2. Laser Wavelengths and Applications for Photoacoustic Effects

### 2.1. Applications at 400–700 nm Visible Wavelength

Applications of photoacoustic technology in the visible wavelength range have been researched for imaging of blood or blood vessels, except when specific substance targets are selected. In the visible wavelength ranges, a 450 nm wavelength laser diode was used for gas detection from nitrogen dioxide (NO_2_) [21] and detection of chromium-doped tetraphenylporphyrin (Cr^III^TPP), and a 532 nm wavelength Nd:YAG laser was used to detect blood flow abnormalities in microvessels [7]. In addition, the application results of photoacoustic technology on perfluorohexane nanoemulsion (PFH-NE) materials and Prussian blue (PB) nanoparticles have been described for treating diseases or contrasts using the absorption spectral wavelengths of specific materials [16].

#### 2.1.1. The 450 nm Wavelength Laser Diode

**Figure 1:**
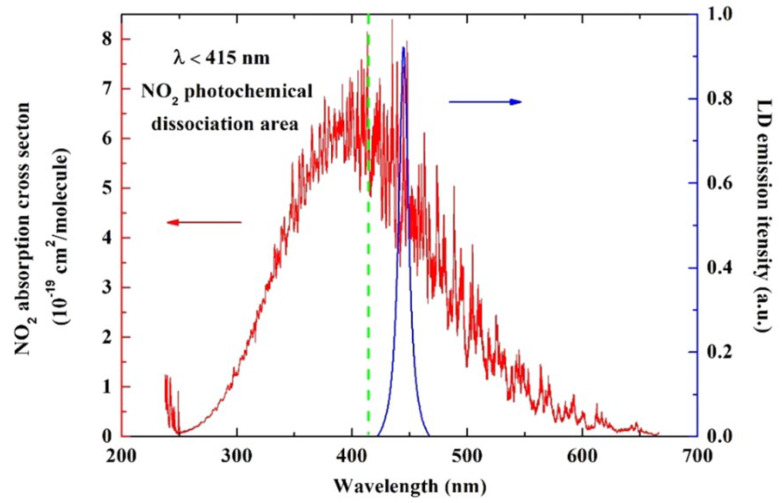
NO_2_ absorption cross section (red) including the photochemical dissociation area (green) and emission spectrum (blue). Reprinted from Li et al., Photoacoustics, 2022, 100325 [21], with permission of Elsevier.

Li et al. from Shanxi University developed a photoacoustic sensor based on quartz-enhanced photoacoustic spectroscopy (QEPAS) technology, which was first introduced in 2002 by modification of photoacoustic spectroscopy (PAS) technology, for one of the major air pollutants, nitrogen dioxide (NO_2_) [21]. A custom blue multimode LD module (Changchun New Industries Optoelectronics Technology, Jilin, China) was used as the laser light source, with a maximum output of 3.5 W in the CW method and a wavelength of 450 nm [21]. As shown in Figure 1, the full width at half maximum (FWHM) was measured as ~7 nm, and the beam quality was improved by reducing the beam divergence angle. The wavelength of the light source was selected in the area with a relatively high cross-section by referring to the absorption cross-section of NO_2_ at 250–650 nm, as shown in Figure 1 [21].

**Figure 2:**
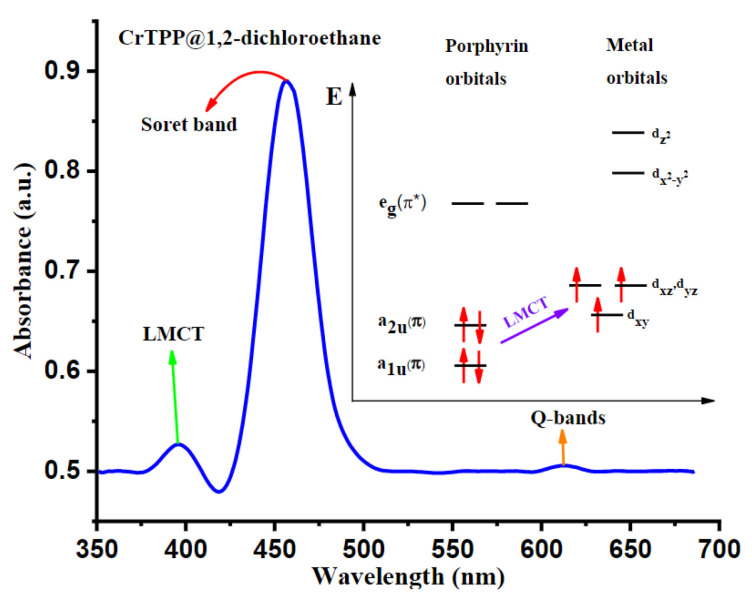
UV–Vis spectrum of the LMCT electronic transition mechanism. Koushik et al., J. Photochem. Photobiol. Chem., 2022, 427: 113811 [7], with permission of Elsevier.

Many systems and technologies using photoacoustic effects have been developed recently and are entering the practical stage. However, the parameters, such as beam characteristics (pulsed or CW), wavelength, pulse duration of the light source, and absorption, which influence the mechanism of photoacoustic effects, have not been studied so far. Koushki et al. conducted a study on the parameters affecting the photoacoustic mechanism and the thermodynamic-dependent photoacoustic effects [7]. This study used a CW laser source to change the intensity around the focus of the beam and to determine the nonlinear photoacoustic effect signal produced in the target substance, a chromium Cr-doped tetraphenylporphyrin (Cr^III^TPP) solution [7]. As shown in Figure 2, the characteristic absorption bands of the ligand-to-metal charge transition (LMCT), Soret band, and Q-band of Cr^III^TPP were used at wavelengths of 400, 460, and 630 nm. As a light source with a wavelength of 450 nm and an output power of 300 mW was used as the CW laser, a focal point diameter of 50 μm, and intensity of 7650 W/cm^2^ were set [7].

#### 2.1.2. The 532 nm Wavelength Pulsed Laser

The laser light source at a wavelength of 532 nm works near the absorption wavelength of hemoglobin, and thus a blood relationship study was conducted [8]. In general, this has been studied using the optical resolution photoacoustic microscopy (OR-PAM) method, which generates many photoacoustic signals of hemoglobin in blood vessels [22] but some studies using the acoustic resolution photoacoustic microscopy (AR-PAM) method have also been conducted for blood vessels in deeper regions [23]. In addition, two or more wavelengths near the hemoglobin absorption peak were used to map the concentration of total hemoglobin and oxygen saturation.

**Figure 3:**
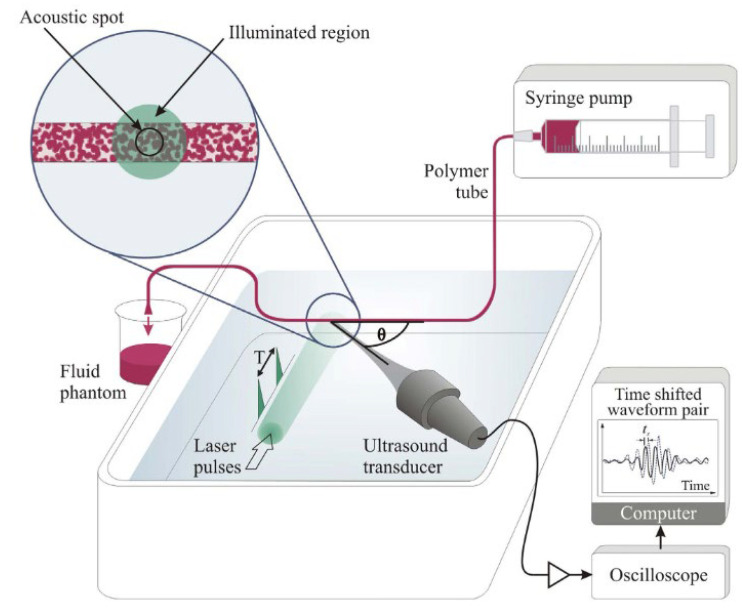
Experimental setup for pulsed photoacoustic Doppler flow measurement. Reprinted from Brunker et al., Sci. Rep., 2016, 6, 1: 20902 [23], with permission of Springer Nature.

In 2016, Brunker and Beard developed the acoustic-resolution photoacoustic Doppler velocimetry technique based on the photoacoustic effects [23]. By overcoming the limitations of conventional blood flow measurement methods and allowing them to be measured at a resolution of several millimeters, it is possible to examine blood flow abnormalities in microvascular vessels (arterial sclerosis, diabetes, cancer, etc.) [23]. The optical microscopy method has a depth limitation (within ~1 mm) for measuring blood flow, and Doppler ultrasound is not suitable for measuring micro blood flow [23]. Photoacoustic flowmetry (PAF) was used to measure the photoacoustic effects on red blood cells. Time, phase, and frequency shifts for photoacoustic signals emitted by red blood cells were measured to calculate the speed of the moving red blood cells [8]. To overcome the limited depth of 1 mm of conventional optical-resolution photoacoustic flowmetry (OR-PAF) method, the acoustic resolution photoacoustic flowmetry (AR-PAF) method was used [8]. In the AR-PAF method, improved blood flow measurement accuracy was obtained using signal processing techniques similar to the range-gating technique utilized in Doppler ultrasound [8]. The experiment was performed with a 3 μm red polystyrene sphere suspension with optical features similar to the size of red blood cells; thus, the measurement accuracy was approximately 1 mm/s, which shows the possibility of measuring the blood flow in a microvascular structure [23]. Figure 3 shows the Doppler photoacoustic flow measurement setup. In the setup, the size difference between the illuminated region and the acoustic spot is shown.

**Figure 4:**
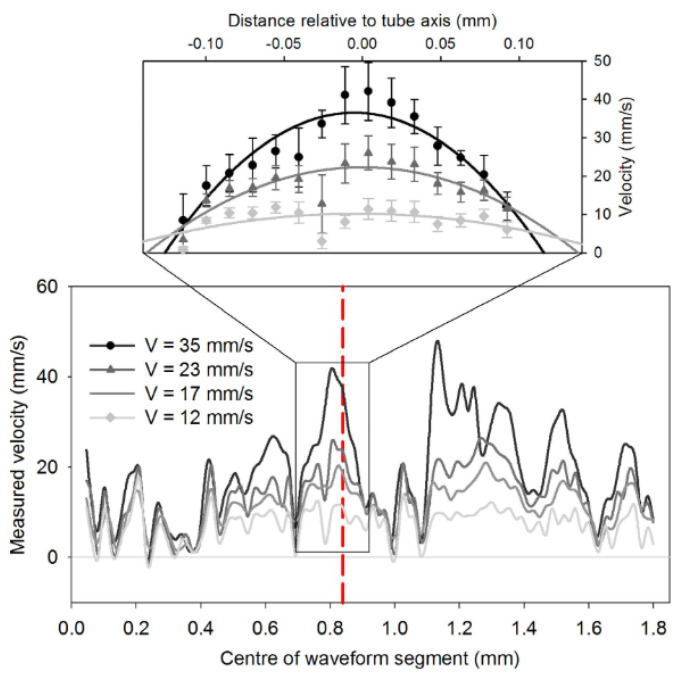
Profiles of velocity measurements corresponding to signal segments. Reprinted from Brunker et al., Sci. Rep., 2016, 6, 1: 20902 [23], with permission of Springer Nature.

Figure 4 shows the speed measurement profile according to the photoacoustic signals. Although it is an AR-PAF method, the shape of the speed profile for the degree of red blood cell size is in position [23]. A pulsed laser (Nano L 200-15, Litron, Rugby, UK) with a wavelength of 532 nm was used as the light source [23]. A pair of optical pulses excite the hemoglobin of the absorber to generate a photoacoustic signal and the time interval between a pair of laser pulses was 0.5 ms [23]. The result showed no poor absorption, although the wavelength of the laser used was not considered as the optimal absorption spectrum (hemoglobin optimal absorption wavelengths of 418, 542, and 577 nm) [23].

**Figure 5:**
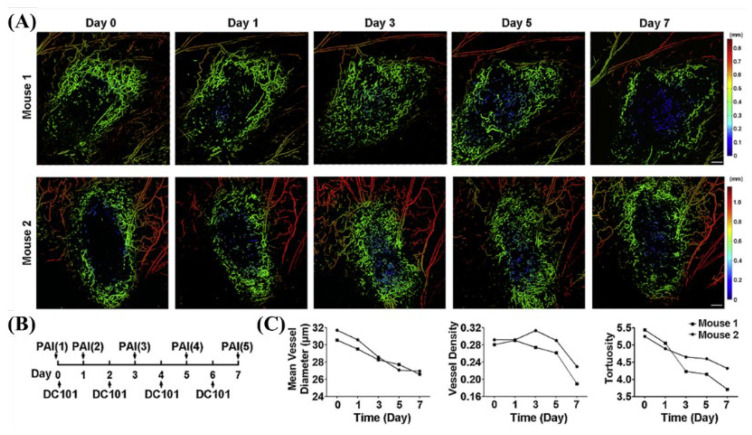
OR-PAM monitored the dynamic changes of tumor vasculature in response to DC101 therapy. (**A**) Depth-encoded maximum amplitude projection (MAP) of tumor vascularity, (**B**) schematic of therapy response monitoring, and (**C**) quantification of tumor vasculature. Reprinted from Zhou et al., Photoacoustics, 2019, 15: 100143 [22], with permission of Elsevier.

In 2019, Zhou et al. developed a scan-type OR-PAM system using a 532 nm Nd:YAG laser (GKNQL-532, Beijing Guoke Laser Co., Beijing, China) and monitored the dynamic changes in the tumor vasculature with an anti-angiogenic agent (DC101-angiogenic agent) therapy [22]. For the photoacoustic system based on a widely used 532 nm nanosecond laser, the wavelength provided sufficient absorption and signal contrast for vascular imaging, and results suitable for monitoring blood vessel formation and changes were obtained [22]. Figure 5 shows the results of calculating the mean vessel diameter, vessel density, and tortuosity as evidence of the treatment effect by monitoring the change in the tumor vasculature with DC101 therapy [22].

**Figure 6:**
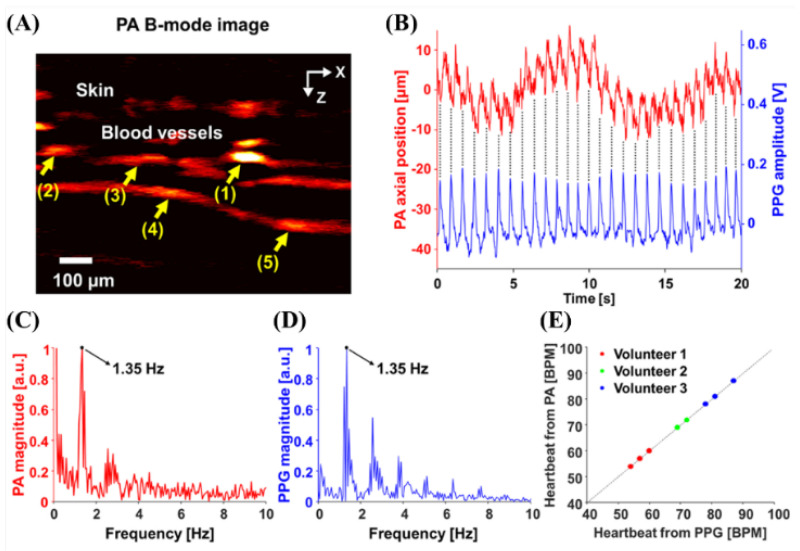
(**A**) Cross-sectional PA B-mode image of a human finger. Quantifications of (**A**) vascular movement. (**C**,**D**) Frequency responses of (**B**) and their dominant frequencies of 1.35 Hz. (**E**) Comparison of the heart rates. Reprinted from J.Ahn et al., Photoacoustics, 2022, 27: 100374 [24], with permission of Elsevier.

In 2022, Kim et al. attempted a study on the photoplethysmography (PPG) system with the explicitness of photoacoustic microscopy of blood vessels in a 532 nm wavelength pulsed laser (AWAVE 532-1W-10K, Advanced Optowave, Ronkoma, NY, USA) [24]. The PPG sensor and PAM signal results were analyzed from data of approximately 60 s, which is more than the actual PPG analysis level (10 s) [24]. In this type of analysis, there are problems, such as the cost and resolution of motion artifacts; however, it is necessary to pay attention to new applications. Figure 6 shows the results of quantifying the changes in PAM vascular motion and PPG signals for five blood vessels.

#### 2.1.3. The 680 nm Wavelength Pulsed Laser

**Figure 7:**
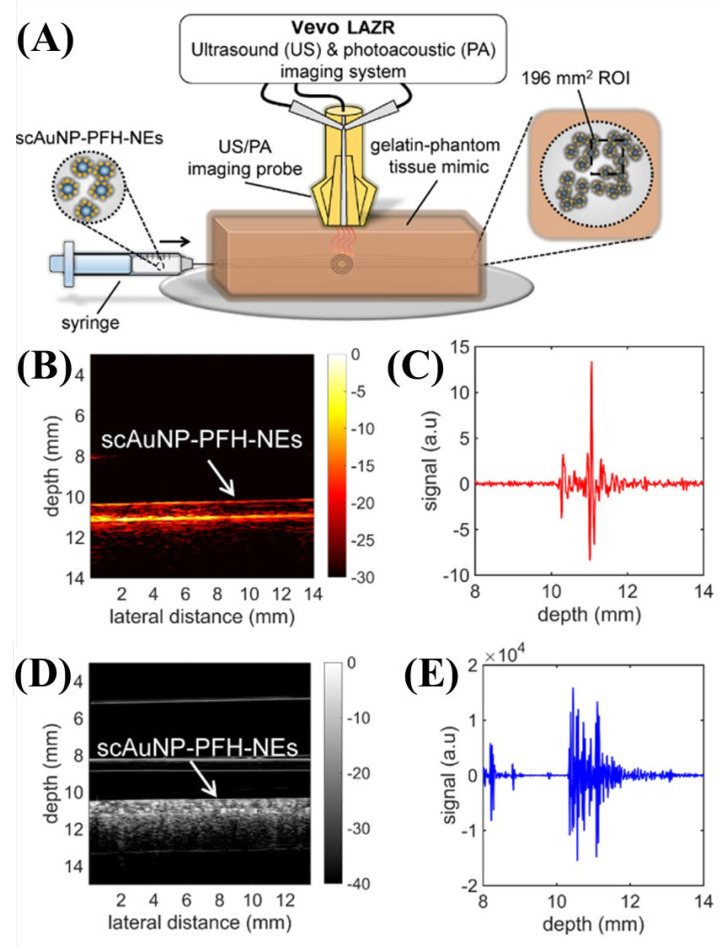
(**A**) Schematic of the Vevo LAZR system setup. (**B**) Photoacoustic and (**D**) ultrasound images of scAuNP-PFH-NEs. (**C**) Photoacoustic and (**E**) ultrasound signals. Reprinted from Fernandes et al., Langmuir, 2016, 32,42: 10870–10880 [25], with permission of the American Chemical Society.

Studies have been conducted on non-invasive ultrasound, photoacoustic imaging, or drug load therapy using a novel drug delivery and contrast medium called perfluorohexane nanoemulsions (PFH-NEs), a nanostructured substance, for cancers in living tissue [25]. In this study, multifunctional imaging techniques, such as photoacoustics, ultrasound, and total internal reflection fluorescence (TIRF), were conducted [25]. In conclusion, local targeting of cancer cells using the novel drug delivery and contrast agent called PFH-NEs was possible, and the strong scattering effect of laser excitation at 473 nm acted as a contrast agent for imaging tumors in photoacoustic and ultrasound applications [25]. The laser used is a commercial LAZR imaging system (FUJIFILM Visual Sonics Inc., Toronto, Canada) equipped with an Nd:YAG laser capable of wavelength adjustment of 680–970 nm [25]. In the experiment, the system specifications were a laser wavelength of 680 nm, a maximum fluence of ~20 mJ/cm^2^, a pulse repetition rate of 20 Hz, and a pulse period of 4–6 ns [25]. Figure 7 shows the experimental results for PFH-NEs and scAuNPs using commercial Vevo LAZR systems.

#### 2.1.4. The 700 nm Wavelength Pulsed Laser

**Figure 8:**
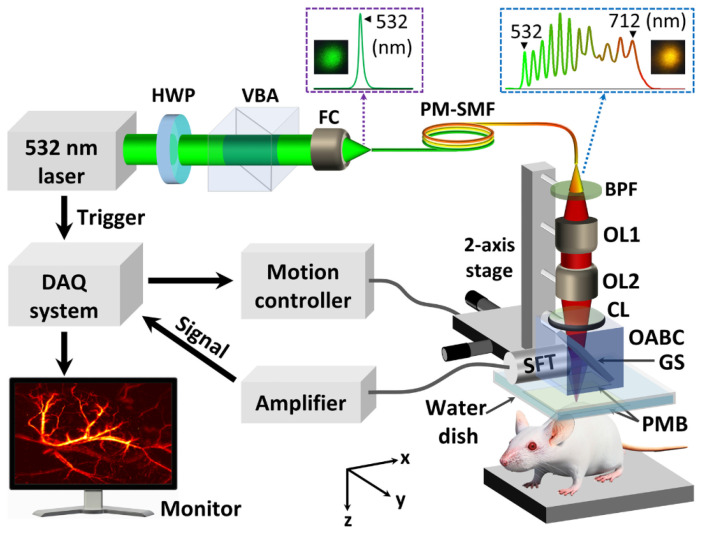
Schematic of tunable-color OR-PAM system. Reprinted from Bui et al., Sci. Rep., 2016, 8,1: 018-20139-0 [26], with permission of Spring Nature.

Contrast agents are widely used for PA imaging. As one of these contrast agents, nanoparticles have been used as a medium in many studies. In addition, nanoparticles can be used to treat cancer or monitor treatments [26]. In 2018, Oh et al. synthesized Prussian blue (PB)-based nanoparticles with a peak absorption at 712 nm in a mouse model with tumors [26]. To monitor this, they developed a stimulated Raman scattering (SRS) light source in the 700 nm region to implement a microscope [26]. As a result, twice the contrast ratio was achieved compared with that of 532 nm light sources, and thus it is expected to become a tool for cancer treatment based on the nanoparticles [26]. The laser was not the expensive OPA or dye laser commonly used for multi-wavelength oscillations, but a tunable-color fiber laser based on the SRS effect [26]. The advantages of SRS-based tunable-color fiber lasers include a wide wavelength of 532–712 nm, a high pulse repetition rate of 50 kHz, and a high pulse energy of hundreds of nJ, suitable for OR-PAM with a relatively low cost [26]. Figure 8 shows the configuration of the OR-PAM system with a tunable color fiber laser.

**Figure 9:**
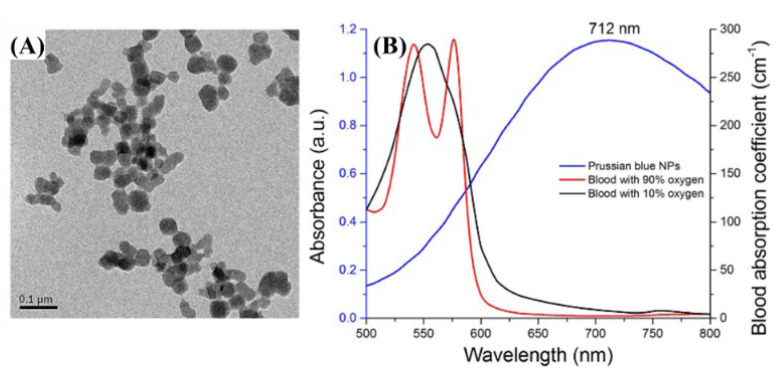
(**A**) TEM image of fabricated PB NPs. (**B**) Measured absorption spectra of PB NPs (blue) and optical absorption coefficients of blood with 90% (red) and 10% (black) oxygen saturation adapted from Prahl 37 and Kollias et al. Reprinted from Bui et al., Sci. Rep., 2016, 8,1: 018-20139-0 [26], with permission of Spring Nature.

The synthesized Prussian blue nanoparticles and absorption peaks are shown below in Figure 9A,B.

**Figure 10:**
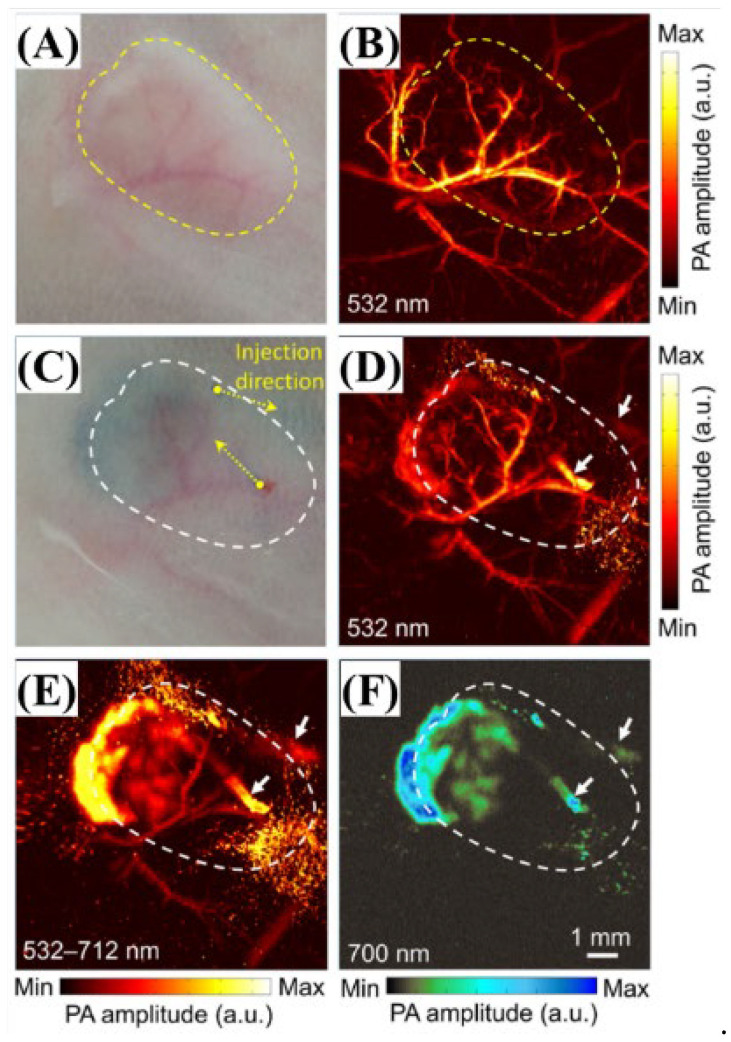
(**A**,**B**) Photograph and corresponding PA MAP image of blood vessels in the mouse tumor model (**C**–**F**). The wavelength and the corresponding pulse energy were as follows; (**B**,**D**) 532 nm, 0.8 μJ; (**E**) 532–712 nm (full spectrum), 1.5 μJ; (**F**) 700 ± 12.5 nm, 0.2 μJ. Reprinted from Bui et al., Sci. Rep., 2016, 8,1:018-20139-0 [26], with permission of Spring Nature.

The results for the mouse tumor models using the OR-PAM technique developed by the research team are shown in Figure 10. Figure 10B,D show the MAP images of the tumor portion at 532 nm and the MAP image at 532 nm wavelengths when PBNP was injected, respectively and Figure 10E,F show the MAP images of the same area using a tunable-color light source, whereas Figure 10F shows the imaging after applying a 700 nm band-pass filter (FWHM of 25 nm) to the light sources [26].

### 2.2. Applications at 800–1600 nm NIR Wavelength

The applications of photoacoustic technology in the NIR wavelength region have been studied to determine whether singularities can be observed at different wavelengths for existing biological tissues, and the characteristics are observed for specific materials according to absorption wavelengths [8]. In the following cases, research studies have been conducted on tumor applications of polyethylene glycol gold nanoshells with maximum absorption at 800 nm wavelength, radiation-damaged nanodiamond (DND) materials with absorption peaks at 820 nm wavelength, semiconductor coopers for cancer treatment with maximum transmission depth at 1064 nm wavelength, and photoacoustic aspects in deep areas [8]. For research purposes, the economic aspects of a pulsed laser diode in light sources at 905 and 1600 nm wavelengths are described.

#### 2.2.1. The 800 nm Wavelength Pulsed Laser

**Figure 11:**
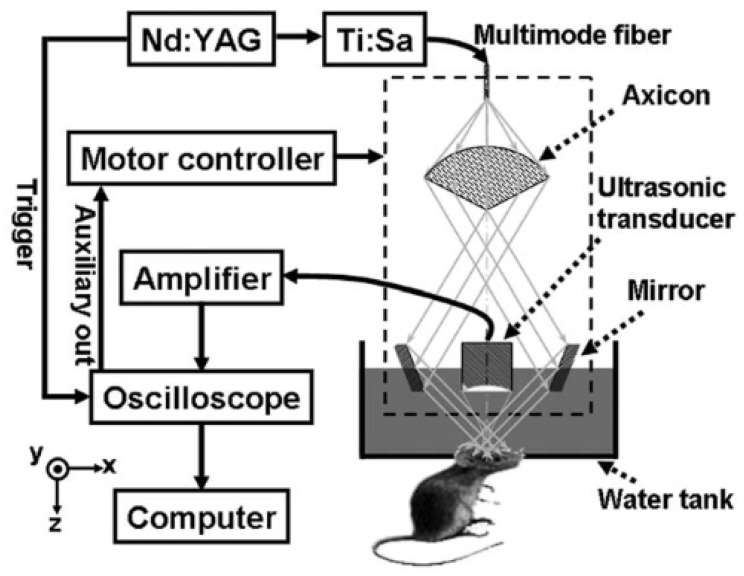
Schematic of the photoacoustic microscope for nanoshell imaging: Ti:Sa, Ti:sapphire. Reprinted from Li et al., J. Biomed. Opt., 2009, 14: 010507 [27], with permission from the Optical Society of America.

Optical imaging technology using contrast medium has high specificity and sensitivity and the size of a gold nanoshell core can be modified by changing its thickness [27]. Thus, the optical properties of nanoshells can vary according to various wavelength ranges (from visible to NIR). Using an 800 nm laser source, Li et al. were able to image the nanoshell distribution in the tumor foci using a PEGylated (polyethylene glycol) injection with maximum light absorption at 800 nm wavelength, which was utilized for thermal therapy [27]. The laser light sources used were a tunable Ti:sapphire laser pumped with Nd-YAG laser, with a pulse width of 15 ns, a pulse repetition rate of 10 Hz, and a wavelength of 800 nm [27]. Figure 11 shows the photoacoustic microscope system configuration for nanoshell imaging research.

**Figure 12:**
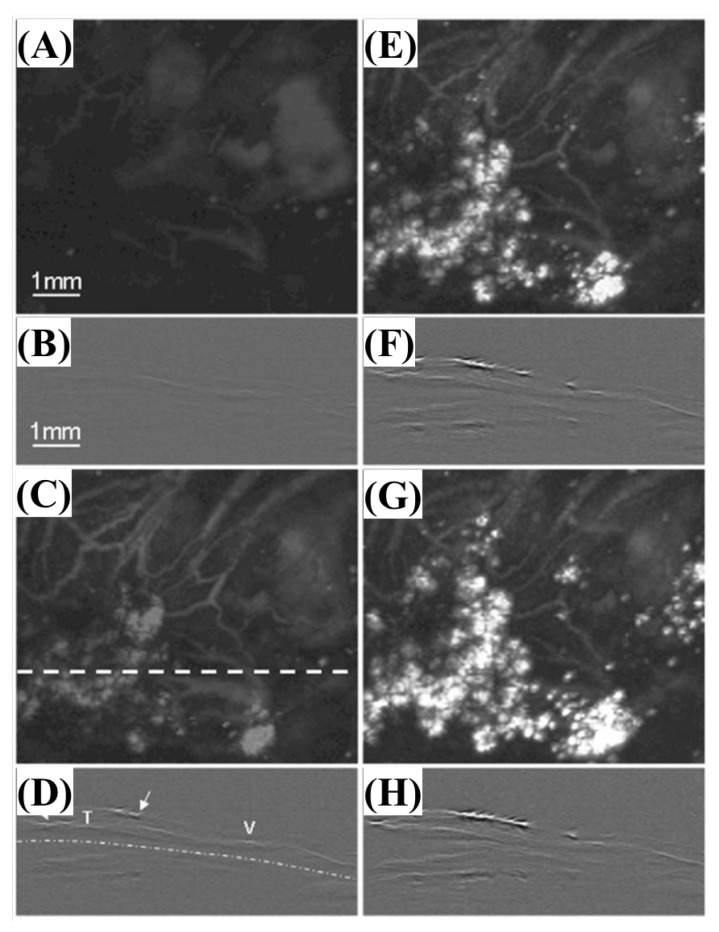
In vivo non-invasive photoacoustic images of nanoshell extravasation from solid tumor vasculature; (**A**,**C**,**E**,**G**) show in vivo maximum-amplitude-projected (MAP) images acquired prior to nanoshell administration; (**B**,**D**,**F**,**H**) show in vivo B-scan images that correspond to the scanning position. Reprinted from Li et al., J. Biomed. Opt., 2009, 14: 010507 [27], with permission from the Optical Society of America.

Figure 12 shows the results of nanoshell extravasation over time after administration into the solid tumor vasculature.

#### 2.2.2. The 820 nm Wavelength Pulsed Laser

**Figure 13:**
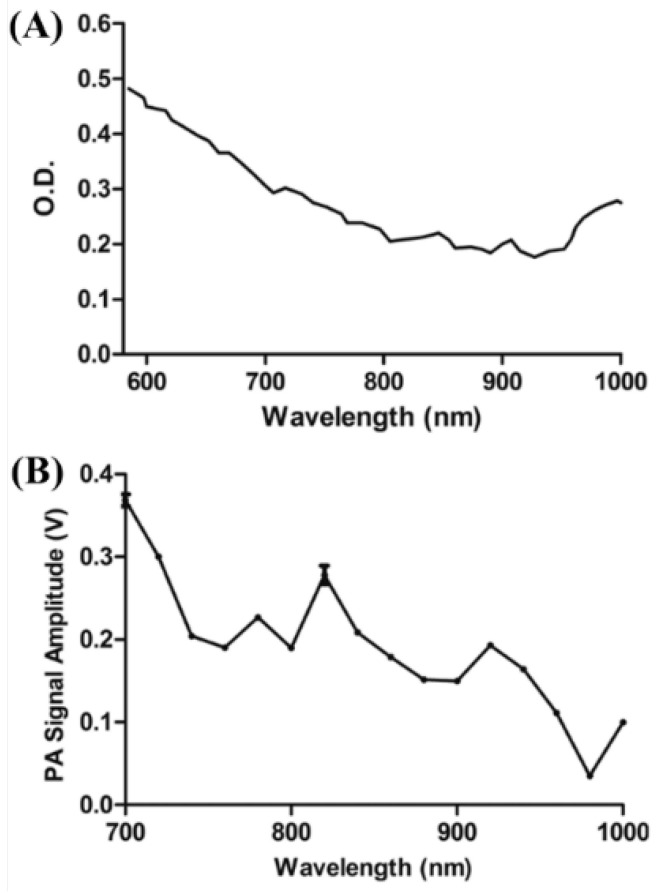
Optical characteristics of DNDs suspended in DI water as functions of the wavelength. (**A**) Absorption spectrum measured with an integrating sphere, and (**B**) PA spectrum. Reprinted from Zhang et al., J. Biomed. Opt., 2013, 18, 2: 026018 [28], with permission from the Optical Society of America.

Zhang et al. used nanodiamonds to perform photoacoustic contrast imaging of living tissues [28]. Although conventional fluorescent nanodiamonds do not have sufficient light absorption in the NIR range, radiation-damaged nanodiamonds (DNDs) synthesized by He^+^ ion beam irradiation have low toxicity and high optical absorbance, making them an ideal contrast medium for biological tissues [28]. The DNDs generated a 71 times higher photoacoustic signal at the same size than the conventional gold nanorods and reported a 5.6 times higher photoacoustic signal at a depth of 3 mm below the skin using an 820 nm wavelength [28]. This is expected to prevent serious disease in some studies of 8–37 nm AuNPs in mice [29]. In addition, the study showed that the size of nanoparticles should be less than 200 nm for cyclic accumulation in leaky tumors and 10–80 nm for absorption and imaging of lymphatic vessels [30]. In this study, a 70 nm diameter DND was used, and the wavelength of the laser was 820 nm, which is the absorption peak wavelength of the synthesized DND [28]. The specification is a 6 ns pulse with a 10 Hz repetition rate, with an OPO laser (Surelite OPO PLUS, Continuum, Santa Clara, CA, USA) pumped with a 532 nm Q-switched Nd:YAG laser [28]. Figure 13 shows the optical properties of the DND. In general, absorption decreased from 590 to 800 nm and then increased near 900 nm, but in photoacoustic signals, 700 and 820 nm were determined in the study to have the maximum and second peaks, respectively.

**Figure 14:**
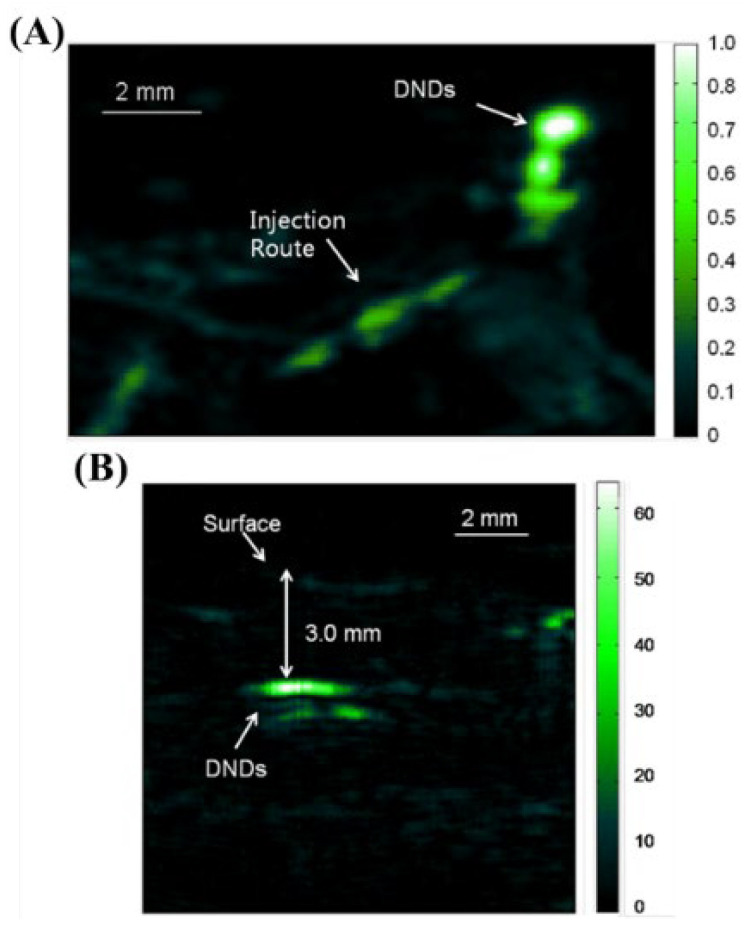
Photoacoustic images taken after injecting DNDs subcutaneously at (**A**) the back (MAP image) and (**B**) the ventral side of the thigh of the mouse (B-scan image). Reprinted from Zhang et al., J. Biomed. Opt., 2013, 18, 2: 026018 [28], with permission from the Optical Society of America.

Figure 14 shows the photoacoustic results obtained after injecting the DNDs into the abdominal side of the thighs of the mouse.

#### 2.2.3. The 905 nm Wavelength Pulsed Laser

In photoacoustic effect applications, the main lasers are Q-switched Nd-YAG, OPO, and Ti:sapphire lasers [8]. However, owing to the problems, such as high price and size, this is an obstacle to the application of photoacoustic effects. To overcome these limitations, Zenget performed photoacoustic imaging in a vessel phantom using a wavelength of 905 nm wavelength, 0.8 kHz pulse repetition rate, and pulsed laser diode [31]. The laser source was a high-power pulsed laser diode array, with a pulse duration of 100 ns. They used the same laser to image carbon fibers, ants, and human hair phantoms of ~4 μm in size [32].

#### 2.2.4. The 1064 nm Wavelength Pulsed Laser

The photoacoustic imaging with a 1064 nm wavelength laser had high contrast and homogeneity for living mouse tissues in the range of 750 to 1064 nm, and up to 70% of background PA signals were low in blood-rich tissues [8]. As the wavelength increases, the change in photoacoustic signals decreases, and more homogeneous photoacoustic signals are provided in vivo over a wavelength of 800 nm [33]. In addition, the 1064 nm wavelength reduces the scattering and absorption of the tissue compared to the lower NIR wavelength, but the energy exposure limitation value increases to 100 mJ/cm^2^ to deliver more light energy [34].

In 2010, Piras et al. conducted a study sequentially infiltrating ductal carcinoma and cyst with a Twente photoacoustic mammoscope using a 1064 nm pulsed laser to address the carcinogenicity of X-rays and the low specificity and high cost of MRI [20]. The prototype photoacoustic application system for mammography is a laser optoacoustic imaging system (LOIS) using a 757 nm NIR pulse and an Alexandrite 1064 nm laser, along with a thermoacoustic computed tomography system (CTT) using 434 MHz RF photons [20]. In this study, they demonstrated that breast tissue has a maximum optical transmission depth in the range of 1000 to 1100 nm, and the light absorption of water and lipids at 1064 nm was minimal [20]. However, in this case, due to the low absorption of hemoglobin, the contrast to the tumor is reduced, so another wavelength, such as 755 nm, is sometimes used (as in a LOIS) [20]. The results of the X-ray, ultrasound, and photoacoustics for breast-infiltrating ductal carcinoma are shown.

In many cases, the contrast agent can enhance the high signal-to-noise ratio (SNR) of the target organization at a wavelength of 1064 nm [16]. Ku et al. used semiconductor copper sulfide nanoparticles (CuS NPs) to study the photoacoustic imaging of deep tissues [35]. For tumor treatment using photoacoustic tomography (PAT), the laser light source and wavelength required for the deep tissue and a light absorption peak near the selected wavelength are needed because the 1064 nm wavelength has little specific absorption, which shows the contrast between tumor cells and normal cells [35]. However, the proposed CuS NPs exhibit sufficient light absorption at 990 nm [35].

**Figure 15:**
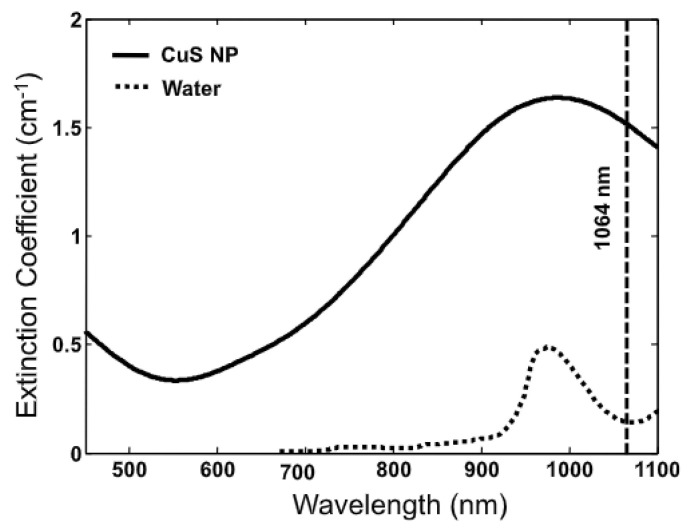
Extinction coefficient spectra of 0.5 mM CuS NP aqueous solution (solid line) and pure water (dotted line). The vertical line is positioned at 1064 nm. Reprinted from Ku et al., ACS Nano 2012, 6, 8: 7489–7496 [35], with permission of the Optical Society of America.

**Figure 16:**
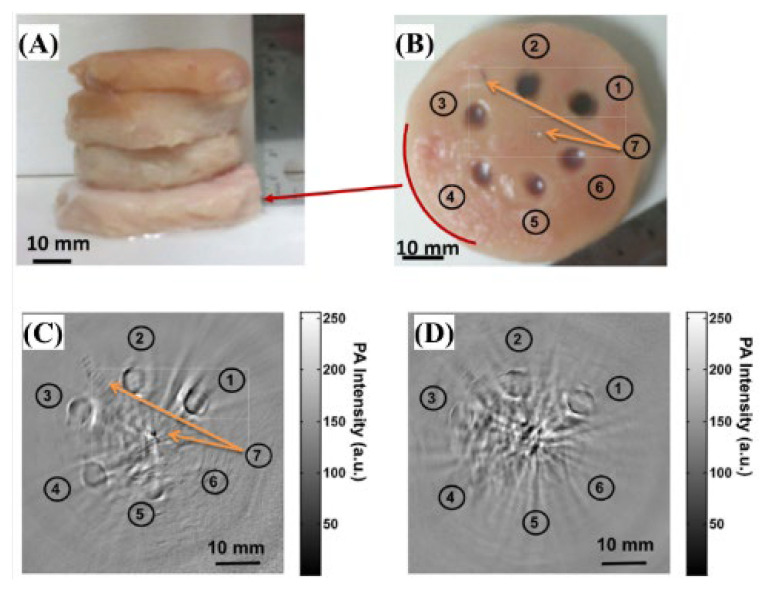
Comparison of deep embedded objects and their photoacoustic images. Photograph of (**A**) chicken breast muscle blocks stacked, (**B**) cross-section of chicken breast muscle with copper sulfide nanoparticles; two-dimensional photoacoustic image at a depth of (**C**) ∼2.5 cm and (**D**) ∼5 cm. Reprinted from Ku et al., ACS Nano. 2012; 6, 8: 7489–7496 [35], with permission of the Optical Society of America.

Figure 15 and Figure 16 show the optical characteristics of CuS NPs at a wavelength of 1064 nm and the difference between the depth and amount of injection. The laser was a Q-switched Nd:YAG laser (LS-2137, LOTIS Tii, Ltd., Minsk, Belarus) with a pulse width of 15 ns and 1 J at 1064 nm wavelength, and ~500 mJ with a repetition rate of 10 Hz at a wavelength of 532 nm [35].

In addition, the human brain was studied using a deep imaging technique at a wavelength of 1064 nm [36]. Although the strong absorption and scattering of the skull’s light and the attenuation and distortion of the ultrasonic waves are generally problematic, Nie et al. used a photon recycler to increase the light transmittance of the skull, resulting in an SNR improvement of 2.4 times compared to the previous one [36]. The laser (Vibrant HE 3151, Opotek, Inc., Carlsbad, CA, USA) is in a range lower than the safety standard set by the American National Standards Institute (ANSI), 40 mJ/cm^2^ at a 1064 nm wavelength, 10 Hz pulse retention rate, and 5 ns pulse width [36].

#### 2.2.5. The 1600 nm Wavelength Pulsed Laser

**Figure 17:**
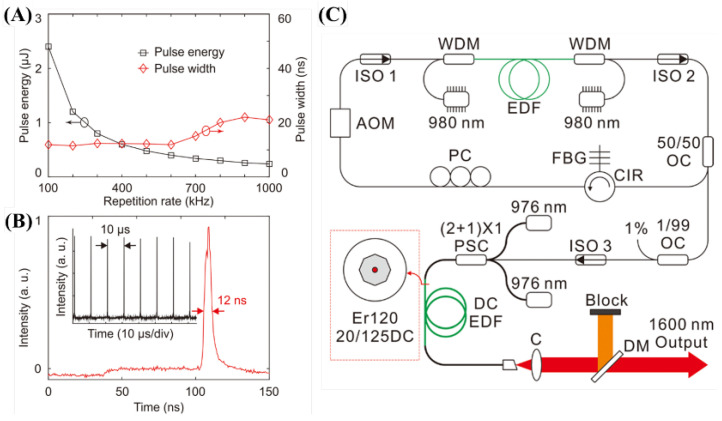
(**A**) Measured energy and width of the output pulse. (**B**) Temporal trace of the output pulse at 100 kHz. (**C**) Schematic of the Q-switched EDFL system. Reprinted from Piao et al., Appl. Phys. Lett., 2016, 108, 14: 143701 [37], with permission of the American Institute of Physics.

To apply photoacoustic effects, it is important to select a target sample suitable for the light sources. However, considering whether the light source or target sample should be considered first, it is important to develop a suitable light source. For research, it is important to have a high wavelength variability through OPO, but it is better to have an appropriate wavelength, pulse width, and energy for price or commercialization purposes. Although it has not been studied extensively in the field of photoacoustic effect applications, Piao et al. developed a light source with a wavelength of 1600 nm for photoacoustic imaging and studied its potential capability for photoacoustic imaging through hair [37]. The developed Q-switched erbium-doped fiber (EDF) laser implements a repetition rate of 100 kHz to 1 MHz, a center wavelength of 1600 nm, a pulse energy of ~2.4 μJ, and a pulse width of 12 ns at 100 kHz [37]. Figure 17 shows the structure, output energy, and pulse width of the developed laser.

### 2.3. Multi-Wavelength Laser Applications

The multi-wavelength method is useful for clarifying the contrast of the specificities of the target tissues or materials rather than using only a single-wavelength laser [8]. We selected the major absorption wavelengths for the target materials and used a wavelength that can express the singularities of the material in the absorption spectrums. In this case study, the imaging study of leukocytes in blood at 532 and 600 nm wavelengths were described; total concentration of hemoglobin, and oxygen saturation (SO_2_) to analyze the circulatory structure and function of microvascular vessels at 532 and 559 nm wavelengths; cerebral hemodynamic changes in response to electrical stimulation at three wavelengths of 560, 570, and 600 nm; endoscopic imaging study of in vivo internal organs combining concentration and oxygen saturation (sO_2_) with ultrasound imaging at 562 and 584 nm wavelengths; cell and tissue imaging studies using tyrosinase (Tyr); imaging studies on plant tissues at 532 nm wavelength integrated with 473 nm CW fluorescence imaging technology; hybrid photoacoustic technology research that fuses multi-wavelength CW laser into a single wavelength pulsed laser to achieve the effectiveness of multi-wavelength photoacoustic imaging studies at low cost; and spectroscopic photoacoustic imaging using molecular vibration [8].

#### 2.3.1. The 532 and 600 nm Wavelength Pulsed Lasers for Leukocyte Imaging

**Figure 18:**
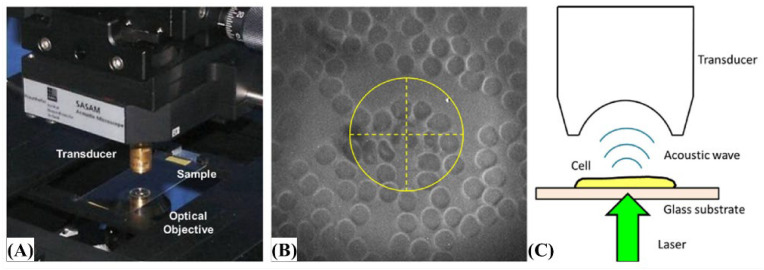
(**A**) Acoustic microscope. (**B**) An optical view of a stained blood smear with the transducer. (**C**) A neutrophil is visible within the crosshairs. Reprinted from Strohm et al., Photoacoustics, 2016, 4, 1: 36-42 [38], with permission of Elsevier.

Conventional ultrasound systems using a 100 MHz transducer are not suitable for single-cell imaging with an axial resolution of 20–50 μm [38]. Kolios developed a micro-resolution system in which 1 GHz ultrasound and photoacoustic systems were combined in the same structure, and attempted to acquire photoacoustic and ultrasonic combined images for blood cell single cells in a blood smear [38]. The laser was a pulsed laser with wavelengths of 532 and 600 nm combined with an optical fiber [38]. However, leukocytes are transparent and difficult to observe without dye [38]. Therefore, they fixed the blood smear as a single layer using a dyed Wright–Giemsa stain (Sigma Aldrich, Burlington, MA, USA) and obtained an image of the leukocytes [38]. This method can identify blood-related abnormalities that are difficult to evaluate using conventional optical microscopy and show detailed structures [38]. Figure 18 shows the structure of the microscopic setup for experiments targeting single cells and obtaining photoacoustic signals.

**Figure 19:**
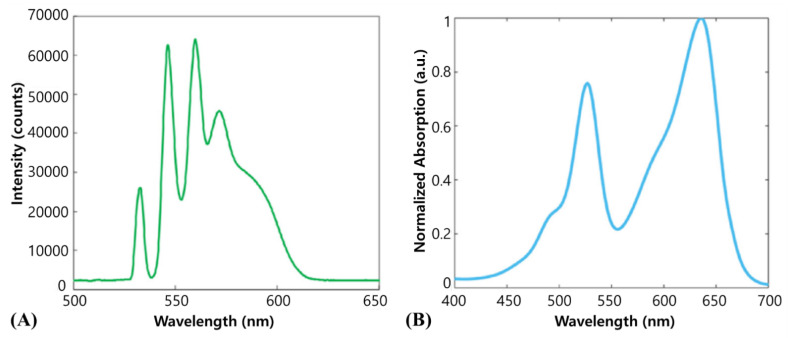
(**A**) Optical wavelengths emitted from the 532 nm fiber-coupled laser. (**B**) The absorption spectrum of the Wright–Giemsa stain. Reprinted from Strohm et al., Photoacoustics, 2016, 4, 1: 36–42 [38], with permission of Elsevier.

Figure 19A,B show the output waveforms that extend the wavelengths by connecting single-mode optical fibers to a pulsed 532 nm laser and the absorption spectrum for light from the Wright–Giemsa stain used for leukocyte staining [38]. The dye shows maximum absorption near 530 and 640 nm, but we believe that the 600 nm wavelength was used because of the laser equipment of the research team.

**Figure 20:**
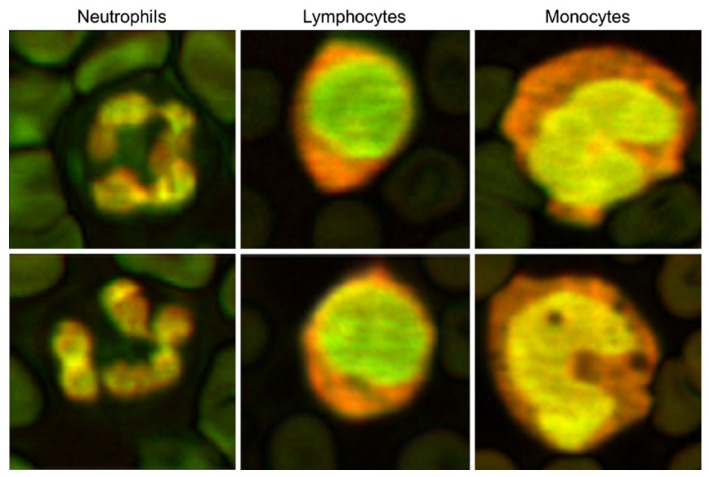
Composite photoacoustic images of the neutrophils, lymphocytes, and monocytes created by merging 532 and 600 nm photoacoustic images. Reprinted from Strohm et al., Photoacoustics, 2016, 4, 1: 36–42 [38], with permission of Elsevier.

Finally, Figure 20 shows cell structures, such as white blood cells, with integrated photoacoustic signals at 532 and 600 nm wavelengths. Therefore, we expect that the photoacoustic effects in single-cell imaging of blood may be useful.

#### 2.3.2. The 532 and 559 nm Wavelength Pulsed Lasers for Oxy- and Deoxy-Hemoglobin Imaging 

**Figure 21:**
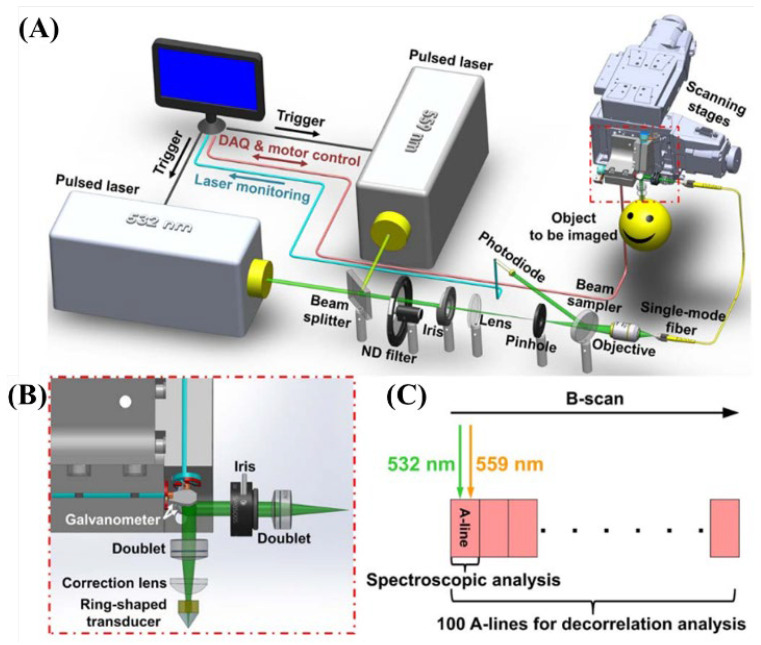
Multi-parametric PAM platform. (**A**) System schematic (DAQ: data acquisition). (**B**) Blow-up of the scan head boxed in (**A**). (**C**) Scanning mechanism for simultaneous acquisition of vascular anatomy, oxygen saturation, and blood flow. Reprinted from Ning et al., Opt. Lett., 2015, 40, 6: 910–913 [39], with permission of the Optical Society of America.

Spectroscopic analysis that distinguishes the properties of the material is possible when two or more wavelengths are used in PAI [39]. Two wavelengths were used to measure the concentration and oxygen saturation of hemoglobin by referring to the light absorption spectrums of oxy-hemoglobin and deoxy-hemoglobin (HbO_2_ and HbR, respectively) in the application of 532 nm wavelength-based photoacoustic vascular imaging [39]. In 2015, Ning et al. mapped and imaged the total concentration and oxygen saturation of hemoglobin up to the capillary level using 532 and 559 nm pulsed lasers [39]. This multi-wavelength vascular research can provide improved techniques for detecting abnormalities in arteries, veins, or microvascular systems by analyzing the structure and function of conventional simple microvascular circulation [39]. As shown in Figure 21, a two-wavelength photoacoustic scanning system was developed using 532 and 559 nm pulsed lasers (BX40-2-G and BX40-2-GR with a repetition rate of 30 kHz, Edgewave GmBH, Würselen, Germany).

**Figure 22:**
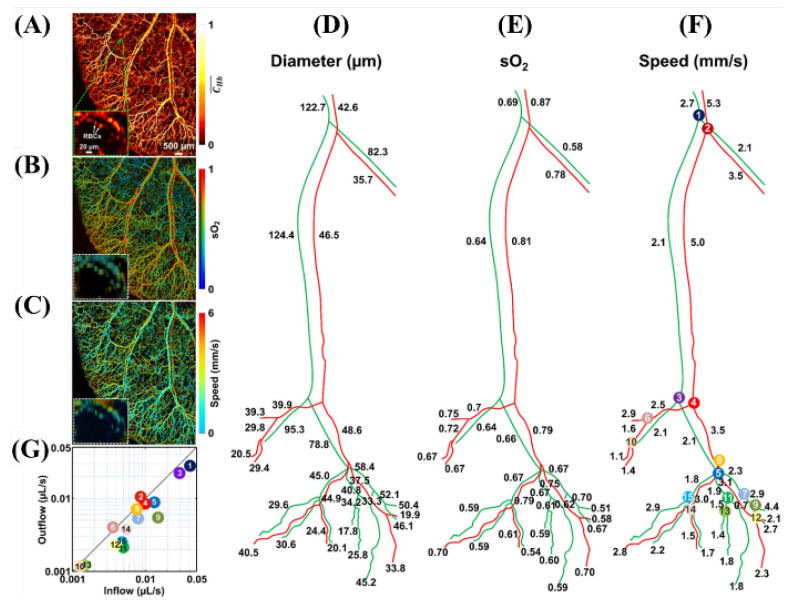
Simultaneous PAM of (**A**) vascular anatomy, (**B**) oxygen saturation of hemoglobin (_S_O_2_), and (**C**) blood flow speed in a nude mouse ear in vivo. Vessel segmentation reveals dynamic changes in (**D**) vessel diameter, (**E**) _S_O_2_, and (**F**) flow speed. (**G**) Conservation of the volumetric inflow and outflow rates at each bifurcation. Reprinted from Ning et al., Opt. Lett., 2015, 40, 6: 910–913 [39], with permission of the Optical Society of America.

As a result of the simultaneous PAM, Figure 22A–E show the anatomical structure of the microvascular system, oxygen saturation of hemoglobin (_S_O_2_), blood flow rate, diameter of the blood vessel, and dynamic changes in the blood flow rate. The use of two wavelengths with respect to the absorption spectrums rather than single-wavelength photoacoustic images can generate a technical advancement of a variety of functional analyses [39].

#### 2.3.3. The 560, 570, and 600 nm Wavelength Pulsed Lasers for Imaging of Rat Brain Hemodynamic Changes 

**Figure 23:**
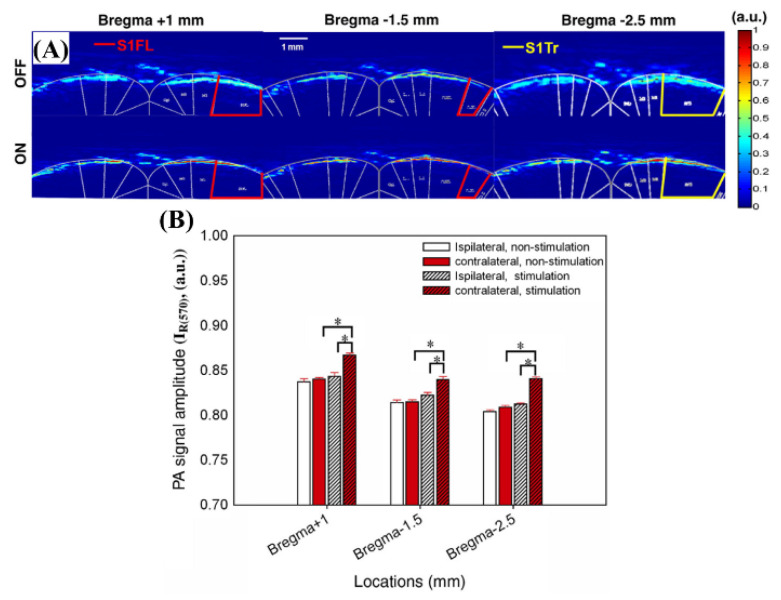
(**A**) In vivo I_R_ (570) images registered and fused with the rat atlas at the positions of bregma +1, −1.5, and −2.5 mm for stimulation-OFF (upper panels) and stimulation-ON (lower panels). (**B**) Quantitative analysis of the I_R_ (570) signal changes in the bilateral ROIs between the stimulation-ON and -OFF conditions. Reprinted from Liao et al., Neuroimage, 2010, 52, 2: 562–570 [40], with permission of Elsevier.

The high contrast to the blood in the photoacoustic system can be used to obtain an image of brain hemodynamic changes to external stimuli [40]. Chen et al. made an image of the hemodynamic changes according to the electrostimulation of the front foot of a rat using an optical parametric oscillator pumped by a frequency-tripped Nd:YAG Q-switched laser at 560, 570, and 600 nm wavelengths [40]. The system was operated with a pulse width of ~4 ns and a repetition rate of 10 Hz. These three wavelengths are areas of high absorption in the blood, and the main photoacoustic signal originates from the cerebral blood vessels [40]. The photoacoustic signals detected at 560 and 600 nm wavelengths are sensitive to sO_2_, and the photoacoustic signal at a wavelength of 570 nm is sensitive to the cerebral blood volume (CBV) [40]. Figure 23 shows the images obtained at 570 nm wavelength with CBV-dominant characteristics, which show the on and off of electrical stimulation and the changes in CBV according to the regions of interest, respectively.

**Figure 24:**
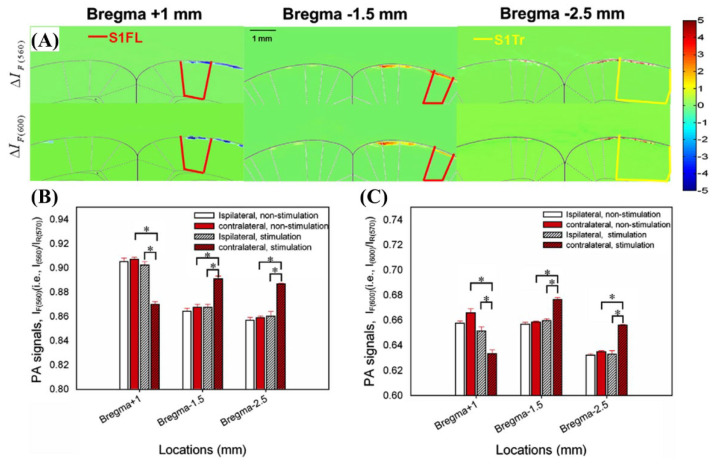
(**A**) In vivo functional ΔI_F(560)_ (upper panels) and ΔI_F(600)_ (lower panels) images registered and fused with the rat atlas at bregma +1, −1.5, and −2.5 mm. (**B**) Quantitative analysis of the I_F(560)_ signal changes in the bilateral ROIs between the stimulation-ON and -OFF conditions. (**C**) Quantitative analysis of the I_F(600)_ in the bilateral ROIs between the stimulation-ON and -OFF conditions. Reprinted from Liao et al., Neuroimage, 2010, 52, 2: 562–570 [40], with permission from Elsevier.

Figure 24 shows the photoacoustic signal results for 560 and 600 nm wavelengths, where the signal changes are sensitive to sO_2_.

In this study, experiments showed that the wavelength most sensitive to sO_2_ variances to stimuli is the photoacoustic signals detected at 560 and 600 nm wavelengths, and they suggested that the signals at 570 nm wavelengths can be oxygenation-insensitive references [40].

#### 2.3.4. The 562 and 584 nm Wavelength Pulsed Lasers for In Vivo Internal Organ Imaging Integrated with Ultrasound Imaging

Unlike ultrasonic imaging, photoacoustic imaging has both functional contrast and high resolution [8]. In addition, when ultrasound imaging and photoacoustic imaging are combined, their advantages are not canceled. Based on this, Wang et al. studied their clinical application for in vivo endoscopy of internal organs [41]. S. K. Nuen et al. developed an endoscopic imaging system that integrated ultrasound and photoacoustic imaging technologies [8]. The ultrasonic images were generated by pulse-echo signals triggered by electric pulses, and photoacoustic images were generated by detecting the acoustic waves generated by laser pulses with wavelengths of 562 and 584 nm [8]. In this study, the distribution of the total hemoglobin concentration and oxygen saturation of hemoglobin (sO_2_) was determined using a pair of photoacoustic signals at 562 and 584 nm wavelengths [16].

J.-M. Yang et al. showed the image results inside the esophagus with radial maximum amplitude projection (RMAP). The total hemoglobin distribution using the 584 nm photoacoustic imaging results, in which the photoacoustic signal corresponding to a wavelength of 584 nm is proportional to the hemoglobin concentration was shown [41,42]. J.-M. Yang et al. showed a sO_2_ image determined from two-wavelength photoacoustic signals and showed the ultrasound imaging results [41,42]. The total hemoglobin distribution and sO_2_ distribution can be imaged using two wavelengths, which were expected to be an application of photoacoustic effects in biological tissues [41,42]. The tunable laser of Wang et al. can freely convert the wavelength and has many degrees of freedom for research based on the optics spectroscopy factor, allowing them to specialize in blood-based oxygen-metabolic application research [41,42].

#### 2.3.5. Beyond 650 nm Wavelength Pulsed Laser for Cells and Tissues Using Tyrosinase (Tyr)

Although the strong absorption of hemoglobin can result in a sophisticated photoacoustic contrast in the vascular structure, most cells and tissues in vivo have relatively low absorption rates at visible or NIR wavelengths [43]. Therefore, imaging is possible using nanoparticles or dye-based contrast agents. Jathoul et al. used genetically encoded reporter genes to study complex biological behaviors while avoiding these restrictions [43]. However, most genetically expressed fluorescent proteins are not ideal for photoacoustic genetic reporters [43]. This is due to the lack of a paucity of photogenic variables above 650 nm wavelength to avoid the absorption of hemoglobin and the low absorbance coefficient [43]. To solve this problem, they proposed tyrosinase (Tyr), a genetically encoded enzyme, as a photoacoustic contrast [43]. It has a strong broadband optical absorption and extends beyond the hemoglobin absorption wavelength with high light stability [43]. A.P. Jathoul et al. showed the system structure used for the in vivo imaging. The laser was a nanosecond optical pulse provided by a fiber-coupled wavelength-tunable OPO [43].

A.P. Jathoul et al. showed the absorption spectra of Tyr, hemoglobin, and fluorescent proteins [43]. The absorption spectrum of Tyr is expressed in K562 cells. A.P. Jathoul et al. showed the results of deep-tissue imaging before and after injection of Tyr-expressing cells obtained at a wavelength of 680 nm [43].

#### 2.3.6. The 532 nm Wavelength Pulsed Laser for Vegetative Tissue Imaging Integrated with 473 nm CW Laser Fluorescence Imaging

**Figure 25:**
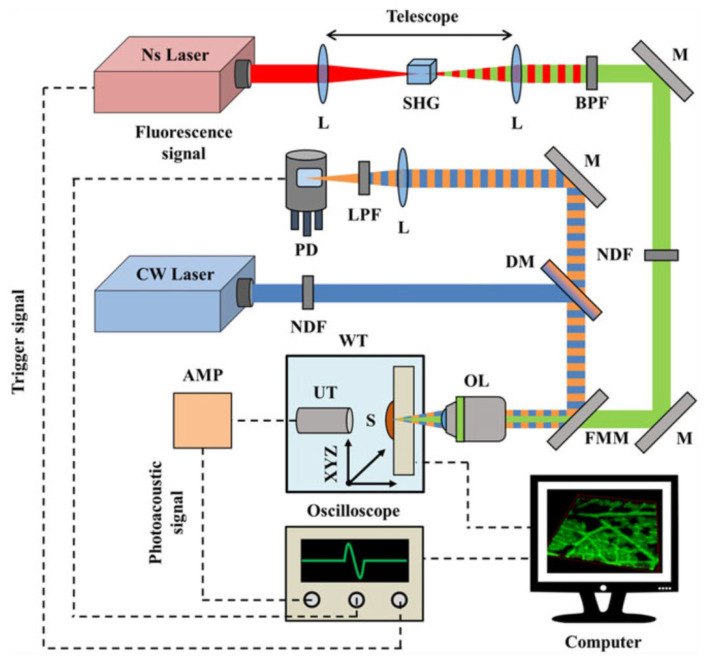
Scheme of the hybrid imaging system. Reprinted from Tservelakis et al., J. Microsc., 2016, 263, 3; 300–306 [44], with permission of Wiley Online Library.

Research on the scientific mechanism of physiology and function of plants, which used to be performed using confocal microscopy with limited depth and fluorescence properties, was conducted on structural images of various vegetable pigments, such as chlorophylls and anthocyanins, through photonic effects with OR-PAM [44]. The system was developed in a hybrid form using optical microscopy to obtain complementary properties for high contrast [44]. Chlorophylls exhibit strong absorption peaks at 428, 452.2, 641.8, and 660 nm, which indicate distinct fluorescence properties in red and NIR light rays [44]. In addition, they strongly reflect in green. Unlike chlorophyll, anthocyanins are mainly responsible for red, purple, and blue colors, such as leaves, flowers, and fruits, and are actively synthesized before, during, and after the fall when chlorophyll decreases [44]. The main absorption wavelength of anthocyanins is 520 nm and the betalain pigment is red and yellow in fruits, flowers, and leaves, with a strong absorption at 535 nm [44]. The laser is a 1064 nm nanosecond pulsed laser with an energy per pulse of 29.4 μJ, a pulse width of 10 ns, a selected repetition rate of 6.8 kHz, and an M^2^ value of ~1.2 (QIR-1064-200-S, CrystaLaser LC, Reno, NV, USA), which produces the outputs at the wavelength of 532 nm through a second harmonic generation crystal (Castech Inc., Fuzhou, China) [44]. This is a strong absorption wavelength suitable for the sample. The laser was selected according to the requirements of the research group. The 473 nm CW LD laser (LSR473U, Lasever, Inc., Ningbo, China; power) with 100 mW and M^2^ value of ~1.2 was used for hybrid fusion with optical microscopes to produce autofluorescence excitation [44]. The developed hybrid system is a fusion system of autofluorescence technology and photoacoustic images, with a maximum lateral resolution of ~3 μm [44]. Figure 25 shows the system structure that fuses a 532 nm pulsed laser with 1064 nm second harmonic generation and a 473 nm laser diode laser for self-fluorescent imaging. The wavelength selection consisted of a band-pass mirror and a flip-type mirror.

**Figure 26:**
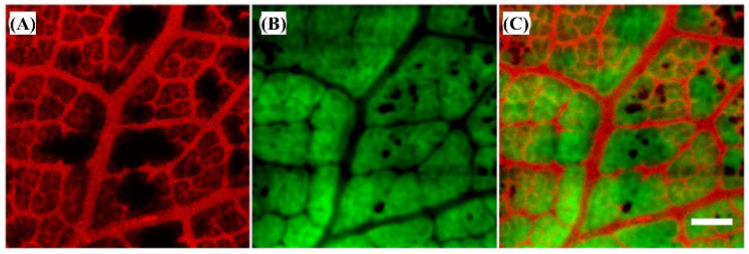
Bimodal photoacoustic and fluorescence imaging of a young rose leaf. (**A**) OR-PAM MAP image depicting the anthocyanin accumulation. (**B**) Chlorophylls autofluorescence image of the same region. (**C**) Combined image of the two contrast modes. Reprinted from Tservelakis et al., J. Microsc., 2016, 263, 3; 300–306 [44], with permission of Wiley Online Library.

Figure 26 shows the results of photoacoustic images for rose leaves and self-fluorescent images, including the combined image results of the two images. This high contrast is expected to help research the growth mechanisms of plants.

#### 2.3.7. Single Wavelength Pulsed Laser with Multiple CW Laser Heating for Photoacoustic Imaging

Multi-wavelength photoacoustic imaging has been extensively researched to study contrast images for different spectral absorptions of biological tissue [8]. In general, multi-wavelength photoacoustic imaging has been achieved using a high-power wavelength variable pulse laser or by combining a single wavelength pulse laser, which has the disadvantage of increasing the system volume and development costs [8]. Hybrid multi-wavelength photoacoustic imaging technology has been used to provide spectral light absorption contrast in photoacoustic images by integrating single-wavelength pulse lasers with multiple CW lasers with different wavelengths without the expensive high-power wavelength variable pulse lasers [45]. This has the advantage of a relatively low cost when using a single wavelength pulse laser and multiple CW lasers, without high-cost light sources such as high-output wavelength variable pulse lasers [45]. However, further research is required to improve the signal against spectral light absorption.

**Figure 27:**
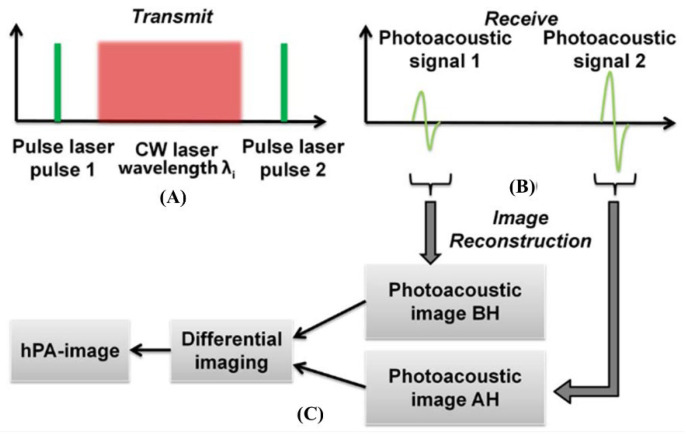
The image reconstruction process of proposed hybrid multi-wavelength PA imaging (hPAI) system. (**A**) Transmitted signal pattern, including two excitation pulses before and after CW laser illumination. (**B**) Received PA signals. (**C**) Image reconstruction process, including PA imaging before and after CW laser heating and hPAI obtained by applying differential imaging. Reprinted from Duan et al., Opt. Lett., 2018, 43, 22; 5611–5614 [45], with permission from the Optical Society of America.

The hybrid multi-wavelength photoacoustic system was constructed by integrating a single wavelength pulse laser of 532 and 1064 nm or other single wavelengths and the multiple CW lasers integrated in parallel with the same optical path [45]. The step motor and pulser-receiver were controlled by a computer to acquire an image through scanning [45]. Figure 27 shows the process for obtaining hPAI results.

#### 2.3.8. Spectroscopic Photoacoustic Imaging

**Figure 28:**
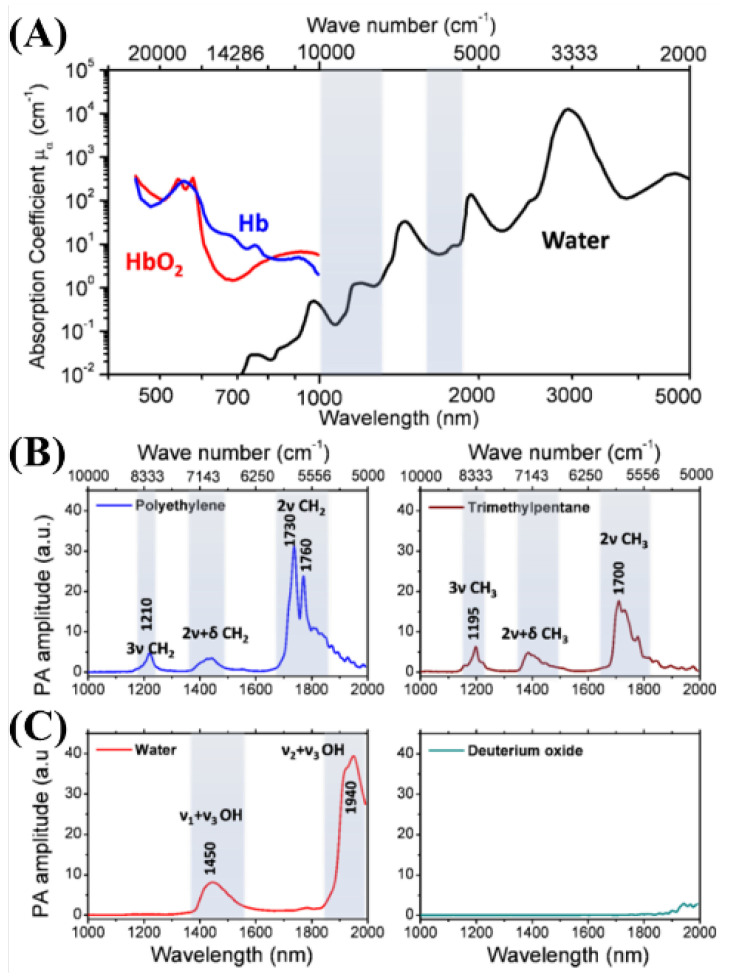
PA spectra of various chemical bond vibrations. (**A**) Absorption spectra of whole blood and pure water. (**B**) PA spectra of polyethylene and trimethylpentane. (**C**) PA spectra of water and deuterium oxide. Adapted from Wang et al., J. Biophotonics, 2012. Reprinted from Wang et al., J. Phys. Chem. Lett., 2013, 4, 13; 2177–2185 [46], with permission of the American Chemical Society.

Vibration spectroscopy and imaging have been used in the biomedical sciences. However, vibration microscopes that apply Raman scattering cannot be used to obtain images of deep tissue [46]. Wang et al. developed a spectroscopic imaging technology for CH bond groups in deep tissues using photoacoustic detection via molecular vibration [46]. They argued that photoacoustic imaging for CH bonds can be more freely utilized in deep tissue problems than that for OH bond groups such as water [46]. In the CH bond group, there are optical windows for the selective imaging of the photoacoustic images, and the main wavelength bands are shown in Figure 28.

**Figure 29:**
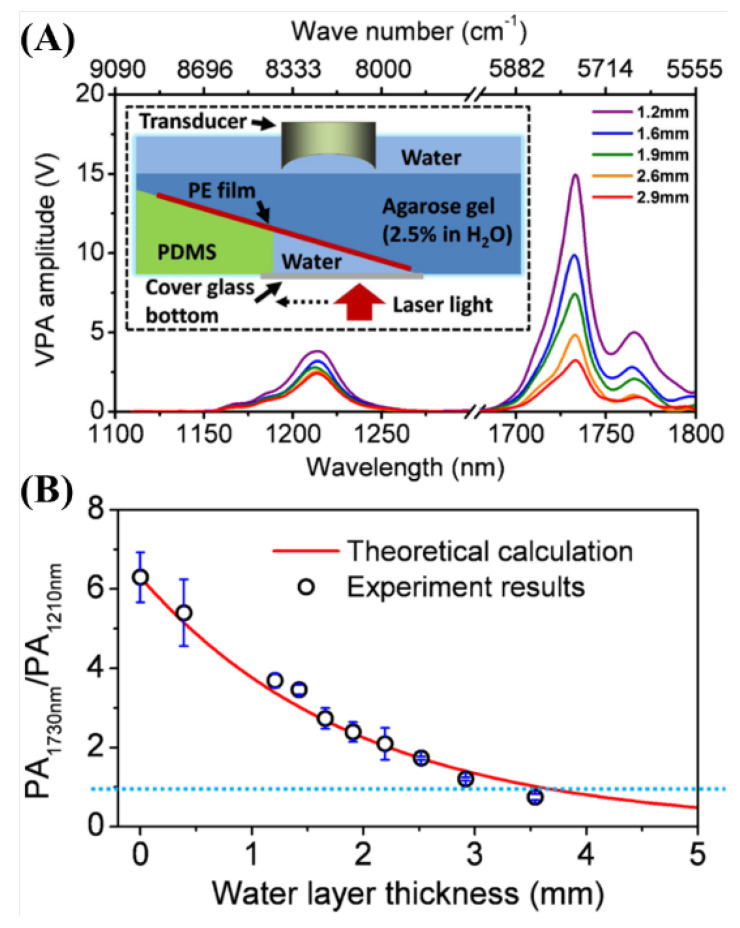
A phantom study that evaluates the effect of water on PA imaging in the near-infrared region. (**A**) PA spectra of PE at different water layer thicknesses. The inset shows a schematic of the constructed phantom. PE: polyethylene. (**B**) PA amplitude ratio between the first and second overtone excitation as a function of the water layer thickness. Adapted from Wang et al., J. Biophotonics, 2012. Reprinted from Wang et al., J. Phys. Chem. Lett., 2013, 4, 13; 2177–2185 [46], with permission of the American Chemical Society.

In addition, they compared the photoacoustic signals according to wavelength and depth to determine which optical wavelengths should be used for selective imaging and demonstrated that a wavelength of 1730 nm obtained beneficial results at 3 mm [46]. The results are shown in Figure 29.

## 3. Discussion and Conclusions

In addition to the preceding wavelengths, laser light sources, such as hybrid optical parametric-oscillating lasers, which exhibit strong absorption of water at 1930 nm, have been developed, but are still in the early stages of finding applications at that wavelength [47]. In addition, a successful noninvasive glucose sensing result was recently reported [48] with single wavelength photoacoustic spectroscopy technology in the mid-infrared wavelength region using quantum cascade lasers (QCL) with high expectations.

We examined and analyzed the applications of photoacoustic technology from visible light to the NIR region, various applications using multiple wavelengths, and the applications of wavelength-specific photoacoustic technology from spectroscopic photoacoustic imaging. Table 1 presents a summary of the laser light sources reviewed in this paper, including references that might be helpful to researchers in finding applications.

Despite the many advantages mentioned earlier, some researchers who are not in the field of photoacoustic technology believe that it is limited to applications in the blood. However, with the efforts of photoacoustic technology, research on the areas beyond the currently limited application fields, including fusion with other imaging technologies, such as ultrasound imaging, applications to internal organs using the endoscopic technique, and use of various contrast agents to develop the photoacoustic technology, have expanded. This proves that photoacoustic technology has the potential to expand the application fields. In addition, most research groups have been conducting research on the optimal absorption wavelengths for the target tissues or materials. In general, such research groups have used tunable lasers, such as OPO, dye, or Ti:Sa lasers, whereas other groups using single- or multi-wavelength lasers have been conducting deep imaging studies close to the target materials or based on the high-energy fluence of the laser. This shows that the infrastructures of research groups are insufficient for research on photoacoustic applications. We expect that this insufficient infrastructure will be gradually resolved with the development of lower costs and more diverse light sources. Therefore, we confirm that photoacoustic technology has significant potential, in particular with the expansion of the research field to optical technology applications.

## Figures and Tables

**Table 1:** Optical source wavelengths for photoacoustic technology.

Wavelength	Pulsed/CW	Model Type	Light Source	Pump Source	Duration	Repetition Rate	Application	Ref.
450 nm	CW			Laser diode		CW	NO_2_ detection (3.5 W), Cr^III^TPP PA(300 mW)	[7,21]
532 nm	Pulsed	532 nm (Nano L 200-15, Litron)	Nd:YAG		6–9 ns	0–15 Hz	Blood flowmetry	[23]
532 nm	Pulsed	532 nm (GKNQL-532, China)	Nd:YAG		-	2 kHz	Tumor DC101 therapy monitoring	[22]
680 nm	Pulsed	680–970 nm commercial	Nd:YAG	-	4–6 ns	20 Hz	PFH-NEs detection	[25]
700 nm	Pulsed	532 nm (SPOT-10-100-532, Elforlight)	Q-Switched Diode	SRS (stimulated Raman Scattering)	1.8 ns	~50 kHz	Prussian blue detection	[26]
800 nm	Pulsed	800 nm (LT-2211A, Lotis TII)	Ti:sapphire	Nd-YAG	15 ns	10 Hz	Polyethylene glycol (PEGylated) gold nanoshell (tumor target)	[27]
820 nm	Pulsed	OPO (Surlite OPO Plus, Continuum, USA)	Nd:YAG	Surelite III, Continuum, USA	6	10 Hz	Radiation-damaged nanodiamond detection	[28]
905 nm	Pulsed	905 ± 15 nm		Laser diode	100 ns	0.8 kHz	Blood vessel phantom	[31]
1064 nm	Pulsed	1064 nm (Brilliant-B, Quantel, France)	Nd:YAG	Brilliant-B, Quantel, France	5 ns	10 Hz	Mammoscope (Breast cancer detection)	[20]
1064 nm	Pulsed	1064 nm, 53 2 nm (LS-2137, Symphotic Tii, USA)	Nd:YAG		15 ns	10 Hz	CuS NPs detection (tumors contrast agent)	[35]
1600 nm	Pulsed	1600 nm (customizable)	EDF	Laser Diode	12 ns	100 kHz–1 MHz	Hair	[37]
532 nm, 559 nm	Pulsed	532 nm (BX40-2-G, Edgewave) 559 nm (BX40-2-GR, Edgewave)			5 ns	30 kHz	sO_2_, flow speed, vessels diameter	[39]
532 nm, 600 nm	Pulsed	532 nm (Teem Photonics, France)		Fiber-coupled wavelength broadening & bandpass filter selection			Leukocytes imaging (1–5 nJ/pulse, 30–160 mJ/cm^2^)	[38]
560 nm, 570 nm, 600 nm	Pulsed	OPO (Surlite OPO Plus, Continuum, USA)	Nd:YAG	Frequency-tripled Nd:YAG Q-switched laser (Surlite II-10, Continuum, USA)	~4 ns	10 Hz	Imaging brain hemodynamic changes	[40]
562 nm, 584 nm	Pulsed		Tunable dye laser (Cobra HRR, Sirah)	Solid-state, diode-pumped, neodymium-doped yttrium lithium fluoride laser (INNOSLAB IS811-E, Edgewave)			Internal organs in vivo	[41]
532 nm(pulsed)473 nm(CW)	Pulsed CW	Variable repetition rate	Nd:YAG LD	1064 nm second harmonic generation	10 ns	6.8 kHz	Chlorophylls, Anthocyanins 29.4 μJ (at 1064 nm)	[44]

## Data Availability

Not applicable.
